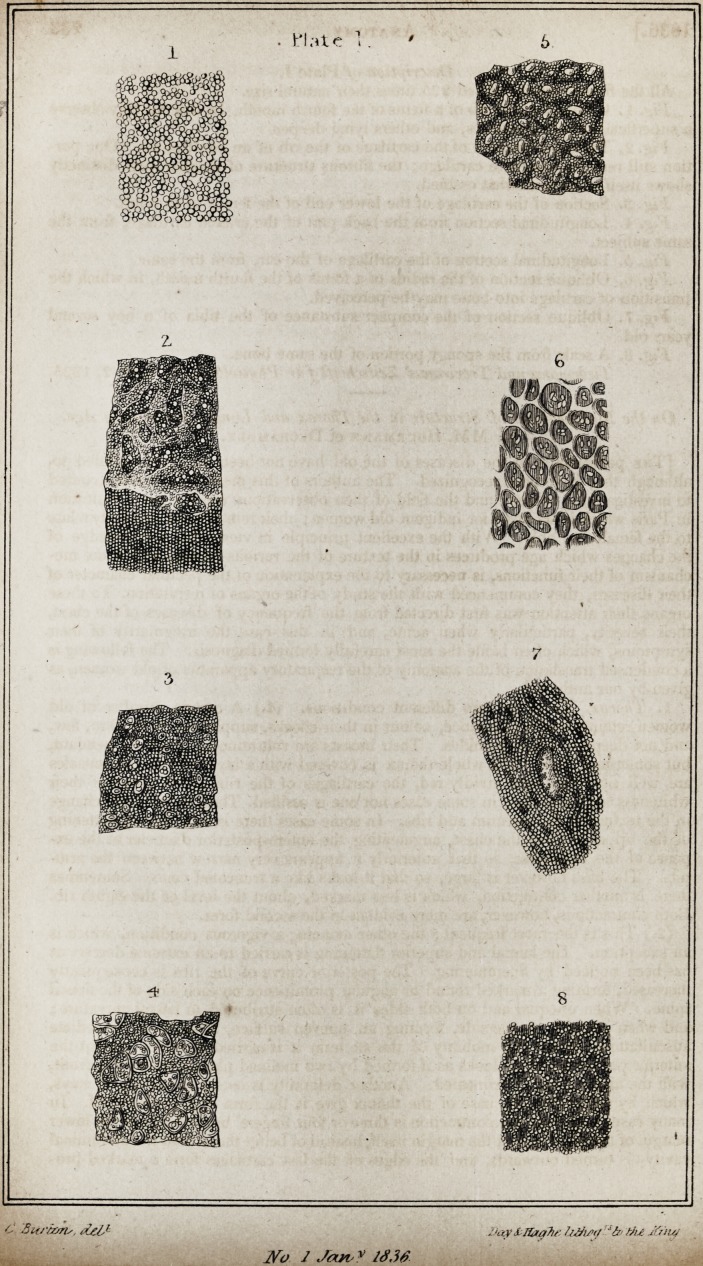# Anatomy

**Published:** 1836-01

**Authors:** 


### PART THIRD.

### Selections from Foreign Journals.

### ANATOMY.

*On the Structure of Cartilage and Bone in the Human Subject.* By W. and F. Arnold.

[The distinguished authors of the present memoir have been long occupied in investigations concerning the texture of the different parts of men and animals, both in the state of health and disease; and, in the paper now before us in the last number of the admirable journal of Tiedemann and Treviranus, they communicate the results which they have obtained respecting the intimate structure of cartilage and bone. The authors inform us that the results as to the structure of bone in adults were obtained by them as early as the spring of 1833. We insert a complete and literal version of this important document, illustrated by accurate copies of the engravings which accompany it in the original.]

1. The true or permanent cartilages in the fœtus of the third, fifth, and seventh month, on the ends of bones, joints, and ribs, consist of numerous granules, apparently of somewhat denser consistency than those in the primary texture of bodies, (bildungsgewebe)*, and placed, for the most part, without any order, near to and over one another.

2. The cartilage from which the bones are subsequently formed, exhibits likewise under the microscope numerous small granules; but these, in particular points, are more closely approximated, so as to leave distinct spaces and tracts between them. These are the more conspicuous, the more advanced the change of the cartilage to bone; but in the cartilages of joints they are less distinct.

3. In those points of the cartilages where the ossification begins, we perceive, on the borders of the bone, the granules united in groups, and distinct spaces between them, which spaces are, for the most part, quadrangular, quintangular, or sexangular. The commencement of the ossification is shown by the appearance in the open spaces of a dark, entangled, arborescent mass, consisting of numerous granules. This and the preceding result we have obtained from comparative experiments on several sections made successively from the cartilage of the joints up to true bone, and taken from the humerus, the radius, the femur, and other hollow bones. By compressing between two glass plates the cartilage which is the matrix of the future bone, and still more, at the period of osseous transformation, we can distinguish a fibrous structure in the separating and disuniting portions; that is to say, fibres are observed to be separated and numerous granules are disjoined, but it is impossible to ascertain with certainty the nature of the fibres, namely, whether they consist of rows of united granules or are tubular.

4. In adults, cartilage appears under the microscope as a white mass composed of granules irregularly heaped together. This mass contains interspaces, which are, for the most part, four, five, or sexangular, but irregularly so; and sometimes they are more of a rounded, oval, or other form. We also observe in them little masses of compressed vesicles of different sizes, of a round or oval shape, and which appear to be, at least in part, adipose vesicles.

5. In the different sorts of cartilage the arrangement is somewhat different. For instance, in the cartilages of joints, the spaces are not always angular, but frequently roundish, oblong, or of no regular form. In the cartilages of the ribs they appear

* We are by no means certain that we have given the exact meaning of this word.

angular, sometimes regular, sometimes irregular, and with four, five, or six angles. In the cartilage of the ear the spaces are mostly oval. The thyroid cartilage appears uniform with that of the ribs, only the interspaces are in the former more rounded and less regular; and we found in the body of a man, forty years of age, here and there in the thyroid cartilage fibrous points, somewhat darker than the rest of the mass; apparently incipient ossifications. The ligamentous cartilages (bandknorpel) consist of numerous granules, constituting a tolerably uniform mass; but, even here, we remark some points more distinct and of a darker colour, arising probably from the stronger or weaker cohesion of the granules.

6. In the bones of adults, treated with dilute muriatic acid, we observe in thin, transverse sections, placed under the microscope, 1. Interspaces of different form and size; 2. Fibres which lie, for the most part, in the same direction as those spaces; 3. Granules constituting those fibres and also contained in the interspaces, in greater or less number, and placed with more or less regularity; 4. Dark masses between the fibres, which appear, and indeed are, by the test of pressure, proved to be formed of minute granules.

7. In the different kinds of bones, the long, short, and flat, and in both the kinds of substance of which they consist, the arrangement of the above-mentioned parts is very different. In the external more compact substance of the hollow bones, the interspaces are round, ovoid, oblong, and also frequently of an irregular shape; consequently the fibres, consisting of rows of granules, (and deriving a jointed appearance from this circumstance,) are, in like manner, observed to have a varying course; and this almost always becomes irregular at the point where the fibres encircling the interspaces unite. This feature is not so distinct in a longitudinal as in a transverse section; in the latter, the spaces appear formed irregularly, and the granules not arranged in rows in a determinate manner. The interior cellular portion of the hollow bones is distinguished from the exterior more compact substance by the circumstance, that, in the former, numerous granules lie round about the individual spaces, and are only in particular spots united formally into fibres. Away from the spaces, the granules exhibit an irregular congeries. In the short bones, and especially in the bodies of the vertebræ, a like configuration is observable. In the flat bones, as in the skull, we perceive numerous spaces of some extent, irregular, or of an oblong form. The bony substance inclosing these spaces is also composed of numerous granules united together. They are less regularly disposed in fibres than in the hollow bones; still, even here we detect an approach to the fibrous structure in the row-like disposition of the granules, although many of them are irregularly heaped together.

8. In the open spaces described we very frequently observe a somewhat spongy or cellular mass, which partly fills them up. This mass consists of numerous granules, which towards its centre are crowded together without any order, but which at the borders of the spaces, as also at the points where it approaches the formal bony matter, are arranged pretty regularly in a fibrous manner, so that there is a gradual transition from this cellular mass (which can be separated from the interspaces, and seems only distinguishable from the true bony substance by its greater sponginess) into the other or proper bone.

9. Cartilages when converted into bone present the same appearances as true bone, for instance, those of the larynx and ribs; as here also we discover many granules, partly united to form fibres, and also some dark points. [Plate I.]

[The authors further state, that they convinced themselves of the existence of granules in the cartilages and bones of the fœtus and adult, not merely by the fact of a quantity of granules being separated, or even the whole mass comminuted into distinct granules, when thin slices were pressed between glass plates;—but also by this, that they could verify the existence of numerous granules in the fluid surrounding the object, of precisely the same diameter (viz. $\frac{1}{400}-\frac{1}{500}$ P.L.—Paris lines ?) as the former. They conclude their memoir by stating that they reserve to a future opportunity the application of the results of the foregoing investigations, to the doctrine of nutrition, the mode of growth of bone, and the conversion of cartilage into bone.]

Plate I.

1

5

2

6

7

3

4

8

C. Burton, delt.

Day & Haghe lithogrs to the King

*Description of* Plate I.

All the figures are magnified 225 times their natural size.

*Fig.* 1. Cartilage of the rib of a fœtus of the fourth month. In this we may observe a superficial series of granules, and others lying deeper.

*Fig.* 2. Transverse section of the cartilage of the rib of an adult of 40. One portion still remains complete cartilage; the fibrous structure of another part distinctly shows itself to be somewhat ossified.

*Fig.* 3. Section of the cartilage of the lower end of the femur of an adult.

*Fig.* 4. Longitudinal section from the back part of the cricoid cartilage, from the same subject.

*Fig.* 5. Longitudinal section of the cartilage of the ear, from the same.

*Fig.* 6. Oblique section of the radius of a fœtus of the fourth month, in which the transition of cartilage into bone may be perceived.

*Fig.* 7. Oblique section of the compact substance of the tibia of a boy several years old.

*Fig.* 8. A scale from the spongy portion of the same bone.

*Tiedemann und Treviranus' Zeitschrift für Physiologie*, B. v. H. 2, 1835.

---

*On the Modifications of Structure in the Thorax and Lungs, produced by Age.*
By MM. Hourmann et Dechambre.

[The peculiarities of the diseases of the old have not been minutely attended to, although they have been recognized. The authors of this memoir have endeavoured to investigate the subject, and the field of their observations was the large institution in Paris which is set apart for indigent old women; their remarks consequently relate to the female sex only. With the excellent principle in view, that a knowledge of the changes which age produces in the texture of the various organs, and in the mechanism of their functions, is necessary to the explanation of the peculiar character of their diseases, they commenced with the study of the organs of respiration. To these organs their attention was first directed from the frequency of diseases of the chest, their severity, particularly when acute, and in this case the irregularity of their symptoms, which often baffle the most carefully formed diagnosis. The following is a condensed translation of the anatomy of the respiratory apparatus of old women, as given by our authors.]

1. *Thorax.* There are two different conditions. (1.) A certain number of old women retain a fresh appearance, colour in their cheeks, suppleness of the skin, few, and not deeply-marked wrinkles. Their breasts are voluminous, generally pendant, but sometimes firm. The whole thorax is covered with a layer of fat, the muscles are well nourished and vividly red, the cartilages of the ribs retain some of their whiteness aud elasticity: in some cases not one is ossified. There is but little change in the texture of the sternum and ribs. In some cases there is some lateral flattening of the upper part of the chest, augmenting the antero-posterior diameter at the expense of the transverse, so that anteriorly it appears very narrow between the armpits. The base however is large, so that it looks like a truncated cone. Sometimes there is another contraction, which is less marked, about the level of the eighth rib. Both contractions, however, are more evident in the second form.

(2.) This is the most frequent; the other evincing a vigorous condition, which is an exception. The lateral and superior flattening is carried to an extreme degree, as has been noticed by Soemmering. The posterior curve of the ribs is consequently increased, forming a marked round or angular prominence on each side of the dorsal spine. When unequal and on both sides it is often attributed to lateral curvature; and when unequal on one side, forming an uneven surface, it impedes immediate auscultation. From the mobility of the sternum it is carried forwards, so that the anterior part of the chest looks as if formed by two inclined planes, meeting in front, with the angle of union truncated. Another deformity is owing to the use of stays, which by contracting the base of the thorax give it the form of a small barrel. In many cases, however, the contraction is three or four fingers' breadth above the lower margin of the ribs: so that the margin itself, instead of being thrust into the abdominal cavity, is turned outwards, and the edges of the last cartilages form a marked prominence beneath the soft parts. This alteration of form is important, from the changes it produces in the viscera beneath. The liver, instead of being always pressed up towards the chest, is, on the contrary, thrust downwards into the abdominal cavity. The portion beneath the stricture is found strongly applied to the upper surface of the liver, which often bears the mark of the pressure that it has suffered on a level with the contraction. The right lung is not pressed up towards the summit of the chest, but is elongated by following the liver in its descent, so that there is not the usual difference in the length and volume of the two lungs. The sternum is forced forward, but as the clavicle and first rib prevent its motions superiorly, the xiphoid cartilage is pressed backwards, and is sometimes overlapped by the cartilages of the last true ribs, which are brought so near together as sometimes to cross each other. (See Cruveilhier's Anatomy, vol. i.) This force, acting in contrary directions at each end of the sternum, produces a kind of separation of the two upper pieces of which it is composed, and an arched eminence at the point where the diastasis takes place. Soemmering has remarked this, as well as the change in the relation of the anterior planes of the thorax and pelvis. They no longer correspond, but the former exceeds the latter; the contrary being the case with old men.

[We have given these details on the effects of stays, from a conviction that the subject cannot be urged too strenuously or too often on the attention of the medical practitioner. From the frequent opportunities which he so particularly possesses, of explaining familiarly, both to the parents and their daughters, the consequences of such unnatural compression, he may often be of great service in diminishing the number of those who are permanently injured by this civilized barbarity.]

The dimensions of the chest are also changed in the longitudinal direction, from a diminution in the height of the intervertebral substance. Fischer mentions the case of a man, æt. 100, in which nine of the vertebræ were reduced to a complete column of bone; and the same change was observed by Boerhaave in the whole length of the spine: our authors have seen very commonly three or four vertebræ thus united. Haller attributed this to the absorption of the intervertebral substance, and Morgagni to its ossification; both admit, however, that the spine is shortened. The absorption is more common than ossification. As the muscles from age become unable to maintain the trunk erect, it bends forwards; and the flat surfaces of the bodies of the vertebræ press strongly against the anterior part of the disks which separate them. In this way (as Seiler observed) union takes place, rendering the inflexion permanent. The last cervical and first dorsal vertebræ are most bent. In some the cervical region makes almost a right angle with the dorsal, and the chin rests against the sternum. A curve in the opposite direction occurs in the lumbar spine, the convexity of which pushes the base of the chest forcibly forwards. From this shortening and curve of the spine, the interval is diminished between the inferior margin of the thorax and spine of the ilium, and also the ribs approach each other more nearly, especially in front. If it is also recollected that from the lateral flattening of the thorax, the ribs are so twisted that their external face is turned directly, instead of obliquely outwards, so that their edges are placed perpendicularly one over the other, the contracted state of the intercostal spaces will readily be conceived. Seiler has also noticed as a consequence of this bend, the elongation of the extensor muscles, and the contraction of the flexors, which contraction becomes permanent; as is seen particularly in the sterno-cleido-mastoidei, which appear like cords when the head is a little raised.

The textures covering the thorax are atrophied; the mammæ and fat have disappeared, and the muscles are very thin. The skin is thin, rough, dry, and of a dirty brown colour. The form of the muscles is seen distinctly. The diaphragm, as may be imagined from so many changes in the form of the thorax, is thrown into folds, which make impressions on the liver. The parts which compose the sternum are united; its cells are large, and often filled with a reddish pulp. The ribs are also less dense, and have lost their elasticity. The cartilages of the first and second ribs are ossified, but it is rare to find bony incrustations on the others; when it takes place it begins in the centre. Union of the chondro-sternal articulations is rarer than is ima gined; the costo-vertebral joints generally preserve their mobility.

2. *Lungs.* The lungs of the old are variable in their aspect. The varieties may be comprehended in three typical forms, by which one lung may differ from another, or parts of the same lung from other parts. Magendie has attended to this subject.

1st Type; or the lungs of muscular, stout, vigorous old women, with a capacious thorax. The external aspect differs very little from the lungs of an adult; except in the direction of the great interlobular fissure, in those cases where there is lateral flatness of the thorax. This fissure in the adult has the upper lobe lying immediately above it, and passes obliquely to the root of the lungs, so that on the right side the central lobe occupies exactly the middle part; and on the left, has the lower lobe immediately beneath it. But in old age the fissure becomes vertical, so that one lobe of the left lung is directly in front and the other behind, and the middle lobe of the right lung projects downwards, and the lower lobe becomes elevated behind it, so as to form the posterior fourth, or even more, of the summit of the organ. Thus pneumonia of the summit may be seated in the inferior lobe.

2d Type. The lungs are of regular form, but small, light, and hardly capable of being distended sufficiently to fill the thorax, even by the strongest inflation. They are bathed in limpid serum. Heart small, thorax contracted, soft parts emaciated.

3d Type. Lungs forming a mass, of which the surface is irregular, pressed close to the spine, and surrounded by much serosity. They are livid and flabby, have lost their conical form, the summit being often larger than the base. The lobes are sometimes merely united by a flat thin pedicle, which leaves them as it were floating: the fissures have disappeared. Inflating them does not much increase their size. They are very light, and give to the touch the sensation of a skein of flax. Heart small, often anæmic; and the thorax often excessively emaciated.

*Intimate Structure.* To examine this the lungs were simply dried, and not previously inflated, for obvious reasons.

1st Type. A thin dried section appeared full of rounded holes, approximating like those in lace. Their diameter was about a quarter of a line, and each was perfectly isolated and distinct. The pulmonary tissue was divided and subdivided very minutely by linear tracts, which were seen by a glass to be evidently vascular. [Plate II. fig. 12.]

2d Type. A similar section was made: the cells were not round but elongated into ellipses, so as to look like a series of chinks, sometimes a line in length, and terminated by two commissures more or less angular. The vascular tracts were equally elongated, and less numerous. The circumferences of the cells were distinctly seen and isolated. [Pl. II. fig. 13.]

3d Type. The cells were of no distinct form: a section could only be compared to torn net-work, whose debris intercepted the irregular spaces. But very few small vessels could be seen by a glass, and there was no division into lobules. [Pl. II. fig. 14.]

Such was the appearance in those who had not had symptoms of disturbed respiration, and so far they may be considered normal. This natural emphysema is more clearly shown when such sections are compared with others from the lungs of adults. In an adult the cells are at least only half the size. In Type 1, the cells were one fourth of a line; in an adult, they were one eighth, or at most one sixth of a line, [Pl. II. fig. 11.]; and in children of four to six years old, about the twelfth of a line, [Pl. II. fig. 10.] finally, in a newly born infant, they were no larger than holes made by the finest needle. These facts verify the law announced by Magendie, from a comparatively superficial examination, that the density of the lungs diminishes, together with the quantity of blood they admit, with the progress of age. The thorax itself is gradually accommodated to this change; it becomes atrophied as the lungs atrophy; it contracts as they contract; and the diminution of their vascularity, which is always in relation to the diminution in texture, shows the direct proportion between the weakened chemical power and the diminished mechanical forces. The effusion of serum may be owing to the thorax being unable to contract beyond a certain point, so that as the lungs still diminish this may fill the vacancy.

[The paper concludes with some criticisms on the opinions of M. Magendie, for which we have not room. The subject is to be continued.]

*Archives générales de Médecine,* Août, 1835.

*On the intimate Structure of the Intestinal Glands.* By Dr. Boehm.

[The observations of modern pathologists, with reference to disease of the intestinal glands, have created a desideratum in the physiology and pathology of those minute organs, which a dissertation recently published at Berlin * appears well calculated to satisfy. It contains the results of a whole year's researches on the subject, in animals of different kinds.]

The glands of Peyer (glandulæ agminatæ, plexus intestinales) appear to have the same structure, though a various form, in all the mammalia. Collected in groups of various sizes, they individually consist of hollow corpuscules with a simple cavity, filled with a semi-opaque fluid when the glands are in a healthy state, but found empty in subjects dying of fever, or other diseases affecting the whole system. The parietes of this cavity are composed of two membranous laminæ, a superficial one resulting from the mucous, and a peculiar one beneath, occupying the submucous membrane, and lining the whole cell or vesicle. The cavity so constituted is not furnished with an excretory duct, for which, however, a morbid black speck or incipient ulceration has frequently been mistaken. Each gland is surrounded by a circle of minute tubes, (corona tubulorum,) opening into the intestine, but closed at the other extremity. In birds, on the contrary, a duct is distinctly visible. The fluid within the above-mentioned cavities differs from mucus by its ready solubility in any quantity of water. It contains innumerable whitish globules, in some respects similar to those of blood. The author believes, with MM. Clarus and Louis, that ulceration of the glands of Peyer originates in inflammatory exudation, occurring in the membrane beneath. *Hecker's Annalen, Erster Band. Zweites Heft*, 1835.

---

## Figures and Tables

**Plate 1 f1:**